# Male and female cichlid fish show cognitive inhibitory control ability

**DOI:** 10.1038/s41598-019-52384-2

**Published:** 2019-10-31

**Authors:** Manuela Lombardi Brandão, Ana Marina Tabah de Almeida Fernandes, Eliane Gonçalves-de-Freitas

**Affiliations:** 1 Departamento de Zoologia e Botânica, Instituto de Biociências, Letras e Ciências Exatas, Universidade Estadual Paulista (UNESP), Cristóvão Colombo, 2265, 15054-000 São José do Rio Preto, SP Brazil; 2 Centro de Aquicultura da UNESP, São José do Rio Preto, SP Brasil

**Keywords:** Evolution, Animal behaviour, Behavioural ecology, Neuroscience, Cognitive neuroscience

## Abstract

Inhibitory control is a way to infer cognitive flexibility in animals by inhibiting a behavioral propensity to obtain a reward. Here we tested whether there are differences in inhibitory control between females and males of the fish Nile tilapia owing to their distinct reproductive roles. Individuals were tested under a detour-reaching paradigm, consisting of training fish to feed behind an opaque barrier and, thereafter, testing them with a transparent one. Fish is expected to avoid trying to cross through the transparent barrier to achieve food (reward), thus showing inhibitory control by recovering the learned detour with the opaque apparatus. Both males and females learned to detour the transparent barrier with similar scores of correct responses, whereas females reached the food faster. This result is probably associated to their different sex roles in reproduction: females care for the eggs and fry inside their mouth (thus requiring a high inhibitory control not to swallow them), whereas males have to stay inside the territory defending it against intruder males, which also demands some inhibitory ability not to leave the spawning site and take the risk of losing it. Furthermore, this evidence of cognitive flexibility can enable social fish to deal with unpredictable interactions.

## Introduction

Inhibitory control is a class of executive functions involving the environmental perception and problem-solving skills which demand high behavioral flexibility in controlling or even inhibiting a prompt behavior, previously learned, in order to achieve a better solution in the future^1,2^. This is considered to be a valuable precondition for refined and highly flexible cognitive skills^1–3^. Effective inhibitory control is used today, for example, to address how complex cognitive processes are selected over the evolution of vertebrates^4,5^, as the task is considered to be highly correlated with intelligence^6,7^ and cognitive competence^8,9^. In this sense, this executive function can highlight differences within species as well.

Executive functions allow individuals to cope with fluctuating environments, by adjusting their behavior to survive. These cognitive operational processes may be more or less flexible according to the environmental demands in which a giving animal is living^10,11^. Therefore, environmental characteristics usually affect the way executive functions are selected^12^ and, within species, females and males may present different cognitive flexibility skills due to ecological roles and demands. Such differences between sexes are well known mainly due to their roles in reproduction, which affect their neural structure and physiology^13,14^. Depending on its sex, an individual will have higher concentrations of specific hormones^15^, neuromodulators^14,16^, or cell receptors^14,16^ which will shape its brain accordingly, and affect executive functions, such as inhibitory control. In fact, several studies have shown that males seem to have a stronger inhibitory control than females (e.g., pheasants^17^, baboons^18^, women in the follicular phase^15^). On the other hand, studies with guppies showed that females seem to be more cognitively flexible than males^19,20^, a characteristic directly related to a good performance in inhibitory control tasks.

In the last years, there was an increase in the number of comparative studies on highly flexible cognitive functions, such as inhibitory control, in mammals and in a few bird species as models. Moreover, four recent studies with fish^3,21–23^ demonstrated that fish are also able to interpret and make decisions concerning inhibitory control. However, most studies regarding cognitive skills in non-human animals have been focusing on male models, since females have hormonal fluctuations along their reproductive periods^24^, which may affect their behavior aside a reproductive context. This idea leads to sparse knowledge on the executive functions and cognitive flexibility of female individuals. Here, we tested the possible differences in inhibitory control between the males and females of the cichlid fish *Oreochromis niloticus* (Linnaeus, 1758), the Nile tilapia, a social and polygynous species, whose males invest in mates through a lek system, while females invest in the offspring by mouthbrooding^25,26^. We hypothesized that females would achieve a better inhibitory flexibility score, since they need to inhibit their feeding propensity not to swallow their eggs and fry guarded inside their mouth. We chose this species because it is a social one, hence it demands cognitive flexibility in order to deal with unpredictable interactions in its social environment, and also because Nile tilapia is a widespread model used for studying several mechanisms related to fish behavior.

**Figure 1:**
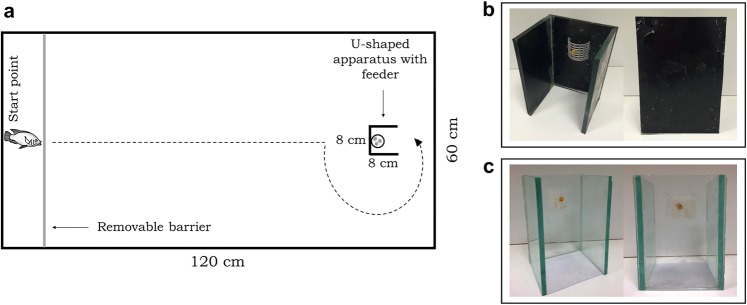
Set up and experimental design. Schematic view of the glass aquarium from above containing the U-shaped apparatus (20 cm high × 8 cm width) and the feeder with shrimp pieces (reward) **(a)**. Fish were individually allocated in the aquarium (120 × 60 × 40 cm) and went through a training phase for 2 days (4 blocks of 4 trials twice a day; 5 min each). After removing the plastic barrier from the start point, fish should perform a detour-reaching task around the U-shaped opaque barrier in order to reach the food **(b)**. Fish that performed 3 out of 4 correct trials in any session of the training phase went on to the test phase (1 day, 2 blocks of 4 trials; 5 min each), in which they should detour a transparent U-shaped barrier to reach the food **(c)**, thus showing inhibitory control by recovering the learned response from the training phase. The U-shaped apparatus was introduced in the aquarium only during the trials, in a way that fish did not need to be removed from their home tanks during the 3 days of experiment. Fish in this figure was draw by A.R. Manzotti and E. Gonçalves-de-Freitas.

Individuals were tested under a detour-reaching paradigm, consisting of training fish to feed behind an opaque barrier and, thereafter, testing them with a transparent one (Fig. 1). Fish should avoid trying to cross through the transparent barrier in order to achieve the reward (i.e., food), thus recovering the learned detour from the training phase and showing inhibitory control. Since the ecological and behavioral roles between Nile tilapia males and females are not similar, we expected to observe different responses in this inhibitory control task and, therefore, in their cognitive functions.

## Results

### Both sexes successfully learned to detour the opaque barrier during the training phase

**Figure 2:**
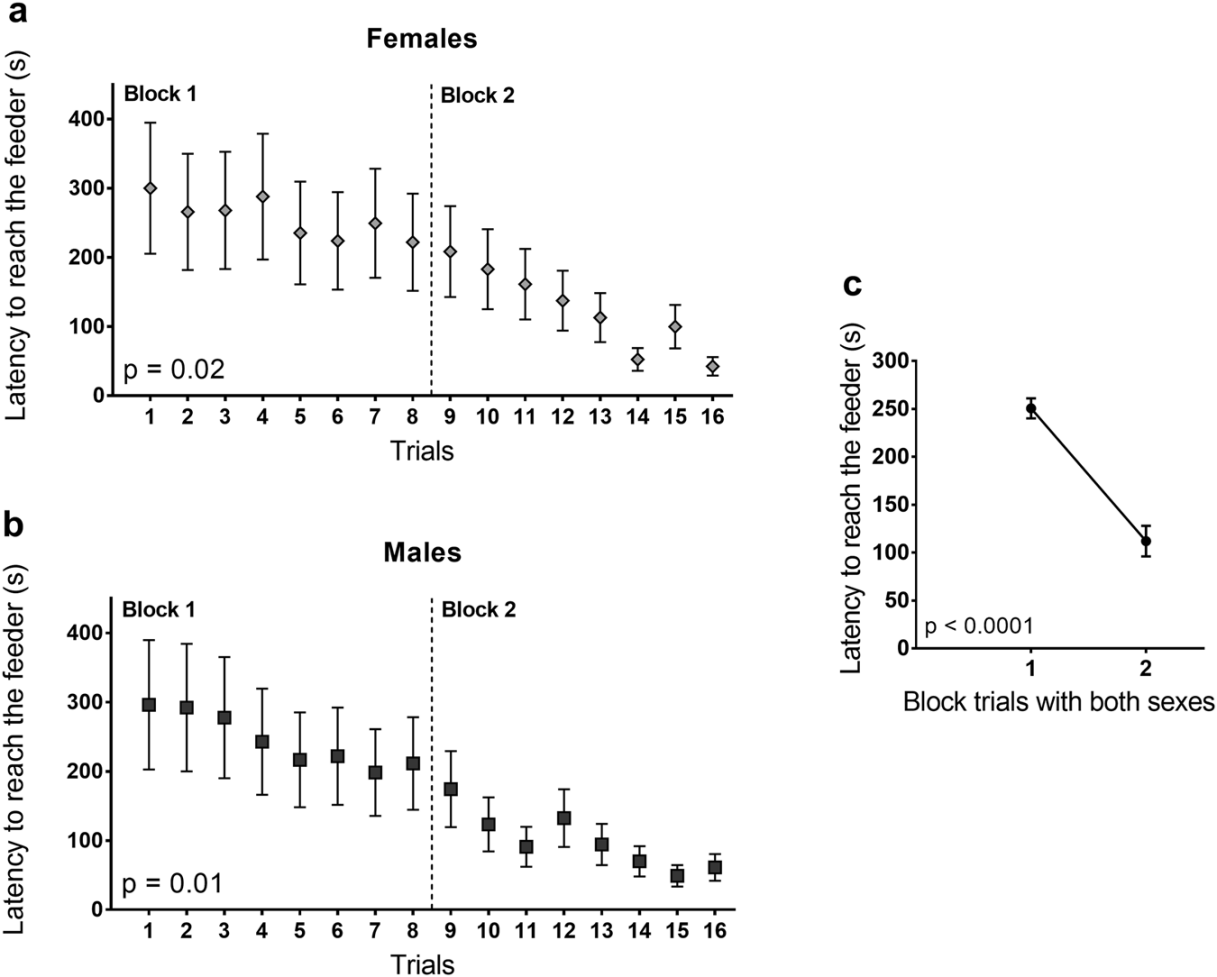
Latency to reach food behind the opaque barrier. (**a)** Females and **(b)** males along the trials in the 2-block training phase (8 trials each block) and **(c)** the total time for both sexes to reach feeder in blocks 1 and 2. LMM completed by Tukey HSD test. Data are shown as mean ± SE.

All subjects met the criterion — 3 out of 4 correct responses — and ate the food when they reached the feeder. Irrespective of the number of correct responses, we also tested latency to reach the feeder by building a learning curve to complete the task with the opaque barrier. This learning curve was built by grouping the first and the second day of training into two blocks of 8 trials each (Fig. 2). There was a significant reduction in the time to achieve the feeder and eat the food from the first to the second block, both in females (LMM, χ^2^ = 4.8; p = 0.02; Fig. 2a) and males (LMM, χ^2^ = 5.7; p = 0.01; Fig. 2b). Latency to reach the food was even lower when both sexes were grouped (LMM, χ^2^ = 4016.4; p < 0.0001; Fig. 2c).

### Males and females showed similar inhibitory control ability

All fish were able to detour the transparent barrier; however, we considered to be a correct response only when fish performed the detour without trying to cross through it. One male individual spent too long to reach the feeder in most of the trials, representing an outlier, and was removed from data analysis. Hence, 70% of females and 70% of males were considered to detour the barrier successfully at least once without trying to cross through it. Variables were analyzed in two blocks (4 trials in the morning and 4 trials in the afternoon), in order to avoid the effect of daily rhythm when comparing males and females. There were no statistical differences between the sexes in the score of correct responses in the morning (GLMM, χ^2^ = 0.006; p = 0.93) nor in the afternoon tests (GLMM, χ^2^ = 0.47; p = 0.49), with an average of 25% ± 2.1% correct responses for females and 29% ± 1.9% of correct responses for males. Likewise, analysis within a same sex showed no differences in the score of correct responses throughout the trials, neither for females (morning: GLMM, χ^2^ = 1.92; p = 0.58; afternoon: GLMM, χ^2^ = 1.5; p = 0.68) nor for males (morning: GLMM, χ^2^ = 2.19; p = 0.53; afternoon: GLMM, χ^2^ = 2.51; p = 0.47).

**Figure 3:**
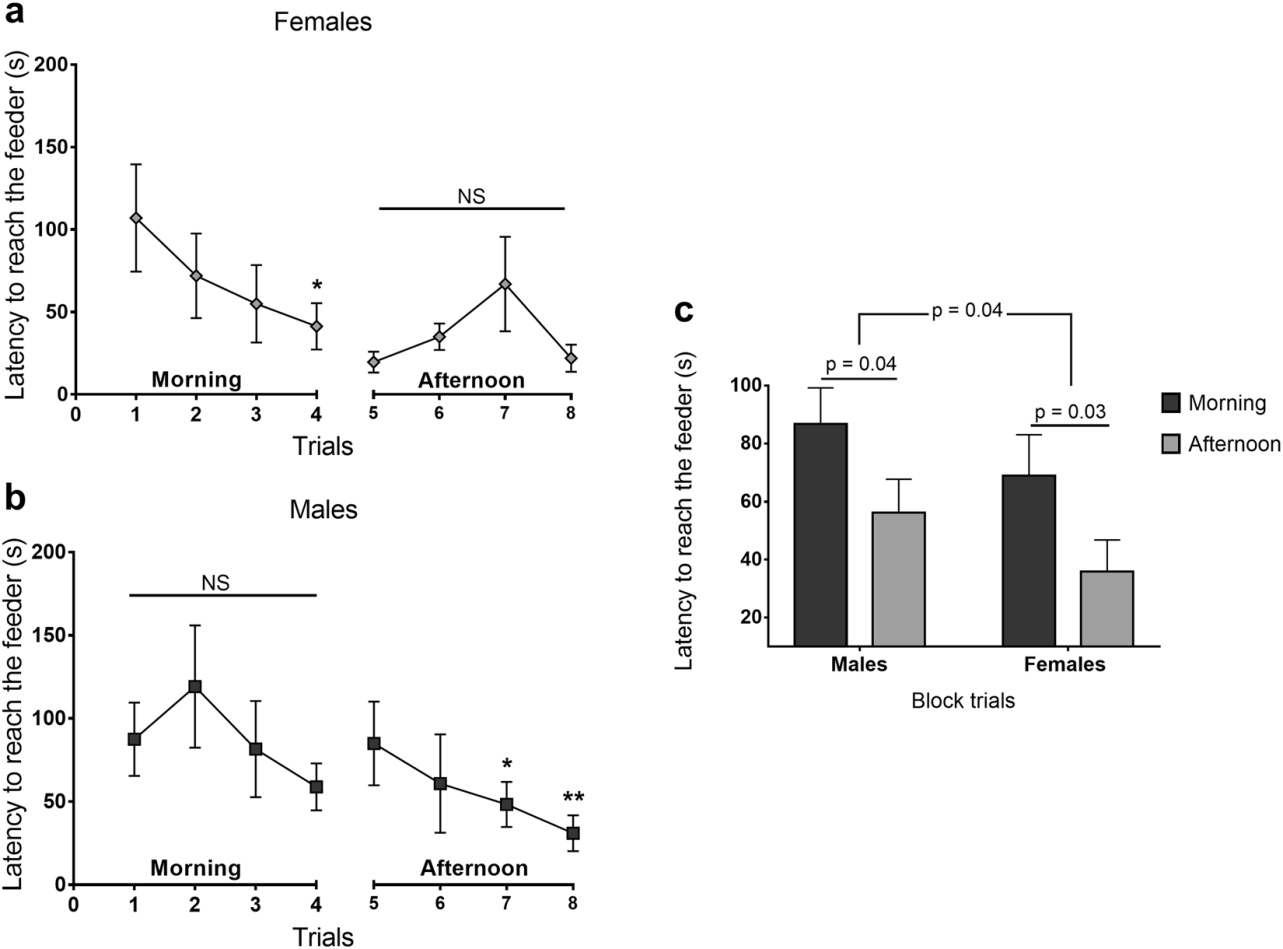
Latency to reach food behind the transparent barrier. Analysis within sex for **(a)** females and **(b)** males, and in **(c)** 2 blocks (morning and afternoon) between sexes. LMM completed by Tukey HSD test after square root transformation. Data are shown as mean ± SE.

Looking at each trial within the sex, latency to reach the feeder for females were already lower in the morning (LMM, χ^2^ = 8.9; p = 0.03) in the 4^th^ trial compared to the 1^st^ one (p = 0.02; Fig. 3a). In the afternoon, there was no significant decrease within the female group (LMM, χ^2^ = 6.43; p = 0.09; Fig. 3a). For males, latency to reach the feeder only decreased in the afternoon (LMM, χ^2^ = 16.12; p = 0.001; Fig. 3b) when the 5^th^ trial (first trial of the afternoon period) was compared with the 7^th^ (p = 0.04) and the 8^th^ (p < 0.001). When compared in blocks of morning and afternoon period, both males (LMM, χ^2^ = 4.13; p = 0.04; Fig. 3c) and females fish (LMM, χ^2^ = 4.67; p = 0.03; Fig. 3c) showed a reduction in the latency to reach the feeder from the morning to the afternoon. Nevertheless, this reduction was more pronounced in females, which is shown by the statistical difference between them and the males (LMM, χ^2^ = 4.19; p = 0.04; Fig. 3c). Overall, the morning and afternoon periods were also different between them, evidencing a reduction in the latency to reach the feeder in the afternoon (LMM, χ^2^ = 9.08; p = 0.003) for both sexes.

## Discussion

We investigated the potential differences between females and males of a polygynous cichlid species, the Nile tilapia, regarding their inhibition against a prepotent response to detour around a transparent barrier by using cognitively demanding functions. Our results indicate that both sexes can perform inhibitory control in a similar way, albeit females seem to learn more quickly than males. We hypothesized that differences between males’ and females’ reproductive roles in the environment could be associated to differences in performing the detour-reaching task. However, both roles require at least some inhibitory control, as discussed below. Furthermore, we showed for the first time that both sexes of a cichlid fish can perform an inhibitory control executive function, similarly to mammals and birds, thus showing some degree of cognitive flexibility.

The detour-reaching task is a commonly used method to infer inhibitory control and, therefore, cognitive flexibility in several vertebrate groups^3,4,27^. However, it is necessary to create conditions in which the cognitive ability is not confounded with other factors in the environment, such as contextual variables^23,28–30^. Thus, although there are some detour-reaching tasks that consist only of detouring transparent barriers, the prevalent methods provide a first training step in which the animal will learn how to detour an opaque barrier before the transparent one^27,31^. This step is important because the majority of animals does not experience “transparent barriers” in their natural environments^29,32^ and the training phase with an opaque barrier makes it easier for the individual to perform and succeed in this task^3,27^. For instance, the training phase provides habituation to the apparatus, thus avoiding bias during the inhibitory control test, such as emotional reactions or other changes in activity arisen from a novel object in the environment^27,28,33–35^. Furthermore, the opaque barrier evidences the motor capacity to perform a detour to reach the goal. Therefore, in our study the training phase reduced the probability of interference in activity due to the novel apparatus in the environment and revealed that fish possess the motor skill to detour it. This type of design is also interesting because it demands the subject to perform a similar response (i.e., detour) as in the previous familiarization trials, although in a different context, with food visible to the animal in the transparent barrier^4^. In this way, we understand that the fish showed some degree of cognitive flexibility because, even though they had already learnt how to swim around a barrier, they had to deal with a new scenario, by using inhibitory control ability. Thus, the behavioral variation presented by males and females Nile tilapia in the test phase was indeed due to individuals’ cognitive abilities rather than to a motor inability or other contextual variable.

Another criticism to the method is the interference of previous experiences with transparency in the environment, such as those found under laboratorial and zoo conditions. In a study with pheasants, individuals that experienced transparency performed better in detour-reaching tasks^36^. On the other hand, a recent study from Santacà *et al*.^23^ with the teleost fish *Poecilia reticulata*, showed that guppies that were raised in transparent tanks performed similarly compared with those without it, thus concluding that there is no effect of that type of previous experience on their detour-reaching task performance^23^. In our study, nevertheless, the subjects were never exposed to transparent barriers besides the transparent apparatus used in the test phase, since all the glass aquaria walls were permanently covered with opaque blue plastic. Therefore, we can assume that our results were not biased by previous experiences with transparent surfaces.

Females and males of Nile tilapia had to reach the feeder with the food reward by detouring a transparent barrier rather than going directly through it and hitting the apparatus walls. Despite the several trials along the test phase, none of the two sexes revealed a significant increase in the score of correct responses, keeping the score near to 25% for females and 29% for males. This is different from other studies, such as those with guppies, which showed a score of 20% and increased it to 58% correct responses. There are several possibilities to explain these differences. For example, the species would behave differently because they are phylogenetic distant and possess different ecological strategies. This hypothesis is plausible, but Luccon-Xiccato *et al*. (2019) showed that fish species phylogenetically distant can show similar performance when tested in similar detour-reaching tasks^37^. Additional explanation could be related to the variation in perceptual and motivational aspects arisen from differences in detour task protocols^29^. For instance, animals tend to perform better and are more motivated in inhibitory control – or detour-reaching tasks – when the goal is a social conspecific instead of food^30^, and when the distance from the goal is shorter^29^ or longer, depending of the context and species^27^. Latency to reach the feeder, however, was reduced both in the training phase, evidencing a learning-curve response, and in the test phase, thus showing that both sexes learned the detour-reaching task. This is in accordance with two other studies — one with guppies^3^ and another one with stickelbacks^21^ — in which fish reduced their time to perform the inhibitory control task along consecutive trials, in spite of the lack of improvement in the correct response scores along them, thus suggesting the need for a learning process on the inhibitory control. On the other hand, studies with birds, such as parrots^38^ and corvids^39^, showed that there was an increase in the correct responses along the trials. It might mean that different groups of animals may interpret inhibitory control tasks differently, probably due to their different brain architectures^27^.

The majority of studies involving detour-reaching tasks are focused on mammals, and they require these animals to reach the reward stimulus by using their limbs (i.e., hands in primates, trunks in elephants^4^, for example). On the other hand, detour-reaching tasks involving fish require them to move their entire body^27^. This “roundabout” principle to reach the reward by moving from a point to another can be more challenging and consequently the cause for the lack of improvement in the scores during the test phase. Likewise, when a fish uses its whole body to detour an obstacle, the risk of exposure in the environment is increased. Therefore, we infer here that it is more difficult for fish to perform such a task than it is for mammals or birds, not only because of their brain architectures, but also because the requirements for testing this task render it more difficult for fish. Thus, considering this scenario, it is plausible to conclude that Nile tilapia were successful in such a detouring task.

In this study, we found no differences between male and female performances regarding the scores of correct responses among Nile tilapia. Nevertheless, their performances were not equal. Nile tilapia males were slower than females to reach the feeder across the trials during the test phase; such difference was already evident during the morning when compared to the afternoon period of test. Several studies, indeed, had demonstrated that females are more prone than males to change their behavioral responses when a learned response becomes inappropriate in a new scenario, thus showing that females are more flexible concerning cognitive demands^19,20^. On the other hand, some studies showed that males are more prone to perform inhibitory control, such as pheasants^17^, chickens^28^, baboons^18^, and even humans^15^. In a study with male and female guppies, Lucon-Xiccato and Bisazza^20^ found out that males were more persistent than females in a previously learnt response, and less able than females to perform a reversal-learning task. The authors suggested that these results were probably due to the different roles of each sex in their natural environment.

According to Kabadayi *et al*. (2018), the results in detour-reaching tasks likely reflect animal’s ecological relevant situations^27^. Thus, the way Nile tilapia males and females dealt with the transparent apparatus might reveal their reproductive differences. Females, for instance, are mouthbrooders and carry their eggs and fry for almost 14 days in their mouth; in addition, free larvae can return to the female’s mouth during a few days after hatching^25,26,40^. In this period of maternal care, females do not feed and must avoid swallowing their offspring. This behavior demands highly inhibitory motor control ability. Males do not inhibit this feeding impulse and will eat the larvae if they can^41^; actually, this is a common type of fry predation among African cichlids^41^. Thus, Nile tilapia females usually care for the young outside the reproductive arena^26,42^. On the other hand, as a territorial and polygynous fish, Nile tilapia males have to show a highly aggressive response in order to be the dominant ones in a territory, chasing away other competitor males and mating more females^26,40,43^. They must stay inside the territories — also their mating site —, which demands some inhibitory capacity in decision-making, thus reducing the risk of losing the spawning site to another male. Furthermore, inhibitory control is not only related to motor skills, but it is considered to be a good method to test cognitive flexibility in animals^4,44^. In fact, it is expected from social species, such as Nile tilapia, to show high cognitive flexibility to deal with the social environment, in which individuals usually face unpredictable interactions^45,46^. Overall, this evidence of cognitive flexibility can be an advantage to Nile tilapia’s reproductive success as well as to its Darwinian fitness.

Besides this type of cognitive ability, several hypotheses (such as the Cognitive Buffer Hypothesis^47–49^ and the Social Brain Hypothesis^45,50,51^) bring the notion that the success in colonizing different environments is directly related to enhanced cognitive flexibility. These theories defend that cognition is a selected factor that allows individuals to be more competent in solving ecological^10,52^ and social^45,51^ problems in order to survive. Thus, animals that have great performance in cognitive tasks are more prone to be successful in new environments^53,54^. Nile tilapia is an example of an ecologically successful species introduced in several types of environments across the world^55,56^. The reasons for this high adaptability is usually explained by its high reproduction rate, fast growth, omnivorous feeding habits, and aggressive behavior^57^, besides its high toleration for salinity fluctuations, temperature variation, and dissolved oxygen levels. Consequently, this species is considered to be a “model invader”^58^. In this study, we showed that Nile tilapia also revealed to be a species with high cognitive flexibility, since both sexes have inhibitory control, a highly demanding executive function, usually found in birds and mammals. Therefore, we can speculate that the success of Nile tilapia in colonizing a wide range of environments may also be due to its remarkable cognitive flexibility, which certainly enables it to cope with environmental novelties.

## Material and Methods

### Fish housing

We used 10 male and 10 female individuals of Nile tilapia, *Oreochromis niloticus* fish from tanks of the university’s fish facility (IBILCE - UNESP, São José do Rio Preto). The fish were gathered from the tanks and taken to the laboratory where they were acclimated for at least 15 days in polyethylene water tanks (ca. 500 L, 1 fish/10 L) at 27 °C and light regime from 7AM to 7PM. Fish were fed once a day to apparent satiation with commercial food for tropical fish (28% CP). Water quality was maintained by using biological filters (400 L/h) and constant aeration.

Before experiments began, animals were allocated in glass tanks (120 × 60 × 40 cm) with gravel and covered walls. Temperature and water quality were maintained under the same acclimation conditions. Our subjects were anesthetized (0.03 g.L^−1^ of benzocaine) to avoid manipulation stress and, subsequently, weighed and measured. Females and males had similar standard lengths (SL mean ± SE: males 12.05 ± 0.25 cm; females 11.71 ± 0.29 cm; unpaired t test, t = 1.23, p = 0.23) and weights (Weight mean ± SE: males = 58.35 ± 3.19 g; females = 53.07 ± 3.14 g; t = 1.53, p = 0.14).

### Experimental design

Nile tilapia males and females were individually tested through a detour-reaching paradigm, consisting of training an individual fish to detour an opaque barrier in order to reach food (reward), followed by a test phase, in which the opaque barrier was substituted for a transparent one. Individuals should avoid trying to cross through the transparent barrier, by performing a detour to reach food, thus showing inhibitory control by recovering the learned response from the training phase. This type of design was based on MacLean *et al*. (2014) that studied 32 species in which subjects were required to inhibit the response that was previously showed in an opaque tube containing hidden food^4^. Then individuals were demanded to perform a similar response (i.e., detour) as in the previous training trials, although in a different context (i.e., with food visible for the animal), thus demanding inhibitory control ability. Lucon-Xiccato *et al*. (2017) used the same approach to test inhibitory control in guppies, using an opaque cylinder in a training phase followed by a test phase with a transparent cylinder^3^. Details are described below, and the set-up is shown in Fig. 1.

### Training phase: the detour-reaching task with the opaque U-shaped apparatus

At the beginning of this phase, subjects were individually allocated in a home glass aquarium of 120 × 60 × 40 cm, filled with 20 cm of water, with gravel at the bottom and walls covered with blue plastic. Blue was chosen due to its effect on reducing the stress in Nile tilapia^59^. Fish were restrained to a space of 13 × 60 × 40 cm by a white plastic barrier at an end of the aquarium (start point) while a U-shaped opaque apparatus containing a feeder attached to it was introduced into the tank (Fig. 1a). The apparatus in this phase was made of glass, covered with a black adhesive to make it opaque (Fig. 1b). The U-shape allows fish to detour it and avoids individuals from reaching the feeder only by chance, perching the apparatus. Note that the U-shaped apparatus was 20 cm high to match the water level, hence preventing fish from reaching the food by going over it.

Fish were trained in two sessions per day, with four trials each session (5 min each trial) at 9AM, 10AM, 11AM, 12PM, and again at 2PM, 3PM, 4PM and 5PM, during two consecutive days (four training sessions total). We introduced the apparatus in the aquarium only during the training sessions. The adopted criterion to go on to the test phase was to reach the feeder by detouring the apparatus and eating the food in at least 3 out of 4 trials. We chose a criterion of 75% correct responses in the training phase in order to avoid prolonged periods of individuals’ isolation during experiments, and to reduce their fatigue and satiation from food reward. The percentage used as a learning criterion is not so different from studies with other species (e.g., 80% correct responses^3,4^). We also compared the latency to reach the food from the moment that the start point’s barrier was removed. This was done on both days of training by forming two blocks of 8 daily trials. Then, we built a learning curve of responses by using the latency to perform the detour-reaching task for males and females (Fig. 2). These both criteria (correct responses and learning curve) allowed that 100% of individuals went on to the test phase.

### Test phase: the detour-reaching task in the transparent U-shaped apparatus

On the third day, we tested the fish in a transparent U-shaped apparatus (Fig. 1c) with the same size and position to that used in the training phase. This similarity is important to avoid a neophobic response from the tested fish towards new objects in this phase^33^, which could impair the developing of the experiment and bias the test (see^34,35^). The test phase showed whether fish were able to recall the same executive function used with the opaque barrier, by detouring the transparent apparatus in order to achieve the reward (i.e., food). Here, fish were tested at the same period as the training phase, in two blocks, at 9AM, 10AM, 11AM, 12PM, and again at 2PM, 3PM, 4PM and 5PM. We quantified fish scores for each trial (i.e., whether fish reached the feeder without trying to cross through the transparent glass wall, it was considered a correct response; in case of an incorrect response, the trial was not repeated). We also quantified the latency that fish took in order to reach the feeder and eat the food from the moment when the start point’s barrier was removed. Since fish promptly learned the task in the 16 trials of the training phase, here we used only 8 trials, assuming that this learning was consistent and stable.

### Statistical analysis

Data analysis was done by using the free software R, version 3.5.1 (http://www.r-project.org), with “lme4”^60^ and “multcomp”^61^ R packages. Data were checked for normality by Kolmogorov-Smirnov’s test and for homoscedasticity by FMax test^62^. Square root transformation was applied for non-homoscedastic data. Those data that did not fit parametric assumptions even after transformation were analyzed by Generalized Linear Mixed Models (GLMMs). Linear Mixed Models (LMMs, “lmer” function of the “lme4” package) analyzed the differences between the two blocks of trials in the training phase and also in the test phase. Scores (correct or incorrect responses in trials) were compared through the GLMMs (“glmer” function of the “lme4” package) and latency to reach the feeder was analyzed also by LMMs. We used Tukey HSD as a post-hoc test (“glht” function of the “multcomp” package). In our models, trials (and sex, when appropriate) were used as fixed factors, and individual identification were used as random effects.

### Ethical statement

This study was conducted according to the ethical principles on animal experimentation adopted by the National Council for the Control of Animal Experimentation (CONCEA – Brazil). It was approved by the Committee on Ethics in Animal Use, UNESP, São José do Rio Preto, permit #143/2016.
